# 肺移植受者肺功能康复管理的最佳证据总结

**DOI:** 10.3779/j.issn.1009-3419.2025.102.34

**Published:** 2025-09-20

**Authors:** Jinhong YING, Ying WANG, Jia QIAN

**Affiliations:** 310024 杭州，浙江大学医学院附属第一医院手术室; Operating Room, The First Affiliated Hospital, School of Medicine, Zhejiang University, Hangzhou 310024, China

**Keywords:** 肺移植, 肺康复, 肺功能训练, 康复管理, 证据总结, 循证医学, Lung transplantation, Pulmonary rehabilitation, Pulmonary function training, Rehabilitation management, Evidence synthesis, Evidence-based medicine

## Abstract

**背景与目的:**

肺移植受者（lung transplant recipients, LTRs）术后的康复管理是影响肺功能恢复的重要环节，本研究系统总结、归纳LTRs术后肺功能康复管理的相关证据，为临床制定LTRs术后肺功能康复管理策略提供依据。

**方法:**

根据“6S”证据模型，系统检索建库至2025年7月，包括UpToDate、BMJ Best Practice、Cochrane Library、Web of Science、中国生物医学文献数据库、中国知网、万方数据库、国际指南协作网、中国医脉通指南网等在内的国内外数据库及网站，提取肺移植术后肺功能康复管理的相关证据，并由2名研究者对文献进行质量评价、证据提取及整合。

**结果:**

共纳入文献18篇，其中专家共识3篇、系统评价/证据总结4篇、随机对照试验4篇、类实验研究5篇、队列研究2篇。汇总最佳证据30条，包括康复评估、早期干预、运动训练、营养管理、药物管理、呼吸功能训练、心理支持及长期随访等8个主题。

**结论:**

本研究基于循证原则总结LTRs术后肺功能康复训练的最佳证据，提出30条临床应用型建议，为临床实施肺功能康复管理实践提供理论依据。临床医护应结合具体情境及专业判断，对证据进行实践转化，为LTRs提供科学的康复管理及指导。

## Abstract (en)

**Background and objective:**

For lung transplant recipients (LTRs), rehabilitation management after lung transplantation is a crucial link affecting the recovery of pulmonary function. This study systematically summarizes and generalizes the relevant evidences on postoperative pulmonary function rehabilitation management in LTRs, thereby providing a basis for formulating clinical strategies for postoperative pulmonary function rehabilitation management in this patient population.

**Methods:**

Based on the "6S" evidence model, a systematic search was conducted in domestic and international databases and websites, including UpToDate, BMJ Best Practice, Cochrane Library, Web of Science, China Biomedical Literature Database (CBM), China National Knowledge Infrastructure (CNKI), Wanfang Database, Guidelines International Network (GIN), and China Yimai Tong Guidelines Network, from the establishment of each database to July 2025. Relevant evidences on postoperative pulmonary function rehabilitation management for LTRs were extracted, and two researchers independently conducted quality assessment, evidence extraction, and integration of the included literature.

**Results:**

A total of 18 studies were included, consisting of 3 expert consensuses, 4 systematic reviews/evidence summaries, 4 randomized controlled trials (RCTs), 5 quasi-experimental studies, and 2 cohort studies. A total of 30 pieces of best evidence were summarized, covering 8 themes: rehabilitation assessment, early intervention, exercise training, nutritional management, medication management, respiratory function training, psychological support, and long-term follow-up.

**Conclusion:**

Based on evidence-based principles, this study summarizes the best evidence for postoperative pulmonary function rehabilitation training in LTRs and proposes 30 clinically applicable recommendations, which provides a theoretical basis for the clinical implementation of pulmonary function rehabilitation management. Clinical medical and nursing staff should combine specific clinical scenarios and professional judgments to translate the evidence into practice, and provide scientific rehabilitation management and guidance for LTRs.

肺移植是终末期肺部疾病患者有效且唯一的治疗方式，对于改善患者肺部功能、提高患者生活质量具有明显的效果，近十年来肺移植数量在全球范围内以每年4000例显著递增^[1]^。随着我国肺移植技术的快速发展和移植中心数量的增加，肺移植已成为终末期肺疾病患者的重要治疗选择^[2]^。然而肺移植受者（lung transplant recipients, LTRs）围术期创伤大、炎症反应多发、术后并发症多，严重制约LTRs术后功能恢复。肺功能训练能协助患者更快恢复胸廓扩张，改善肺功能，减少术后不适症状，降低医疗费用支出^[3]^。且目前国内外虽对LTRs围术期管理已有相关指南及专家共识，但对肺功能康复管理的内容尚欠聚焦。本研究旨在通过循证方法对LTRs肺功能康复管理的相关证据进行总结，以期为临床制定更科学的LTRs肺功能管理方案提供循证依据。

## 1 资料与方法

### 1.1 问题确立

依照PIPOST模式确立循证问题，即证据应用的目标人群（population, P）为LTRs；干预措施（intervention, I）为LTRs的肺功能康复管理，包括评估、训练、指导等；应用证据的人员（professional, P）为临床医护人员；结局指标（outcome, O）为肺功能、运动能力、生活质量等；证据临床转化场所（setting, S）为医院、社区、居家等；证据类型（type of evidence, T）为临床决策、指南、系统评价、证据总结、专家共识及相关原始研究。

### 1.2 文献检索策略

基于“6S”循证资源金字塔模型，自上而下依次检索有关LTRs术后肺功能康复管理的相关证据，包括UpToDate、BMJ Best Practice、Cochrane Library、澳大利亚乔安娜布里格斯研究所（Joanna Briggs Institute, JBI）循证卫生保健中心数据库、国际指南协作网（Guidelines International Network, GIN）、英国国家卫生与临床优化研究所网站（National Institute for Health and Clinical Excellence, NICE）、苏格兰院际指南网（Scottish Intercollegiate Guidelines Network, SIGN）、美国国立指南库（National Guideline Clearinghouse, NGC）、加拿大安大略注册护士协会网站（Registered Nurses' Association of Ontario, RNAO）、Web of Science、PubMed、Embase、CINAHL、国际心肺移植协会网站、中国医脉通指南网、中国生物医学文献数据库、中国知网、万方数据库、维普数据知识服务平台。

中文检索词：“肺移植/肺脏移植/肺器官移植/移植肺/术后肺移植”，“康复/肺康复/肺功能康复/康复管理/呼吸训练/运动训练/肺功能锻炼/体力活动/康复训练/自我管理/居家康复/呼吸肌训练/物理治疗”。

英文检索词：“Lung transplantation/Pulmonary transplantation/Lung graft/Post lung transplant/Lung allograft”“Rehabilitation/Pulmonary rehabilitation/Respiratory rehabilitation/Exercise training/Physical activity/Physical therapy/Respiratory muscle training/Self-management/Home-based rehabilitation/Breathing exercise”。

检索策略示例：以PubMed为例，具体检索式为：（“Lung transplantation”[MeSH] OR "Lung transplant" OR "Pulmonary transplant"）AND（“Rehabilitation”[MeSH] OR "Rehabilitation" OR "Pulmonary rehabilitation" OR "Exercise training" OR "Respiratory training"）AND（“Evidence-based practice”[MeSH] OR "Guideline" OR "Consensus"）。各数据库间采用AND连接，同义词间采用OR连接。

检索建库至2025年7月的高质量文献。

### 1.3 文献纳入与排除标准

纳入标准：（1）研究对象为LTRs；（2）研究内容涉及肺移植术后肺功能康复；（3）研究类型为指南、专家共识、系统评价、荟萃分析、高质量随机对照试验等中英文文献；（4）同一主题的多篇规范、指南、专家共识等参照最新版。排除标准：（1）主题不符：非肺移植术后肺功能康复相关研究；（2）研究对象不符：非肺移植患者或混合其他疾病患者且无法单独提取肺移植患者数据；（3）研究内容不符：仅涉及手术技术、免疫抑制等非康复管理内容；（4）信息缺失、无法获取全文；（5）会议论文、研究计划、报告书、指南解读及翻译类文献；（6）重复发表的文献，文献质量低的文献，包括指南评价结果为C级或研究方法存在明显缺陷的其他证据类型。

### 1.4 文献质量评价标准

UpToDate来源的临床决策作为高质量文献直接纳入，指南通过临床指南研究与评估系统II进行评价，相关专家共识如《病毒性肺损伤所致呼吸衰竭患者肺移植评估专家共识》^[4]^和《肺动脉高压诊断与治疗指南解读》^[5]^等纳入评价范围，专家共识和原始研究采用澳大利亚JBI循证卫生保健中心评价工具（2016）进行质量评价，采用Cochrane偏倚风险评估工具对随机对照试验进行评价。

### 1.5 证据的提取、整合与评级

证据提取采用标准化数据提取表，包括研究基本信息、干预措施、结局指标、证据内容等。证据整合过程采用主题框架分析法，首先由2名研究者独立提取证据条目，然后根据内容相似性进行归类，形成初步主题框架。通过多轮讨论和专家咨询，最终确定8个主题类别。

证据分级采用JBI 2014版FAME系统，具体标准为：可行性（Feasibility, F）评估证据在临床环境中的实施可行性；适宜性（Appropriateness, A）评估证据与临床问题的匹配度；有意义性（Meaningfulness, M）评估证据对患者和临床实践的意义；有效性（Effectiveness, E）评估证据的临床效果。A级证据（强推荐）：在FAME 4个维度中至少3个维度评价为高质量；B级证据（弱推荐）：在FAME 4个维度中2个维度评价为高质量；C级证据（不推荐）：在FAME 4个维度中少于2个维度评价为高质量。

对于多个来源证据结论不一致的情况，处理原则为：（1）优先采用系统评价和*meta*分析结果；（2）其次采用多中心随机对照试验证据；（3）专家共识和指南证据作为补充；（4）单中心研究证据权重最低。最终形成的30条证据中，A级证据18条，B级证据12条，C级证据未纳入最终推荐。

文献筛选过程中，2名研究者独立筛选的一致性采用*Kappa*检验评估，*Kappa*值为0.85，表明一致性良好。由2名研究者独立对纳入文献的作者、发表年份、文献类型、文献主题等基本信息进行独立提取整合，并用JBI循证卫生保健中心的证据推荐级别系统（2014版）对提取内容进行分级，依据FAME属性将证据划分为A级（强推荐）、B级（弱推荐）。若出现多个来源的证据结论不一致则遵循高质量证据优先、循证证据优先、权威文献优先的原则进行评定。若出现2名研究者评价意见不一致，则与第3名循证小组成员进行探讨，最终达成一致。

### 1.6 统计学分析

采用Excel 2025版进行分析。计数资料采用例数（率）表示。

## 2 结果

### 2.1 文献检索结果

通过文献检索共获得中英文文献3256篇，通过去重及筛选全文，删除无法获得数据、文献质量过低等文献，最终纳入18篇文献。其中专家共识3篇、系统评价/证据总结4篇、随机对照试验4篇、类实验研究5篇、队列研究2篇。文献筛选流程见图1，纳入文献的一般特征见表1^[1-18]^。

**图1:**
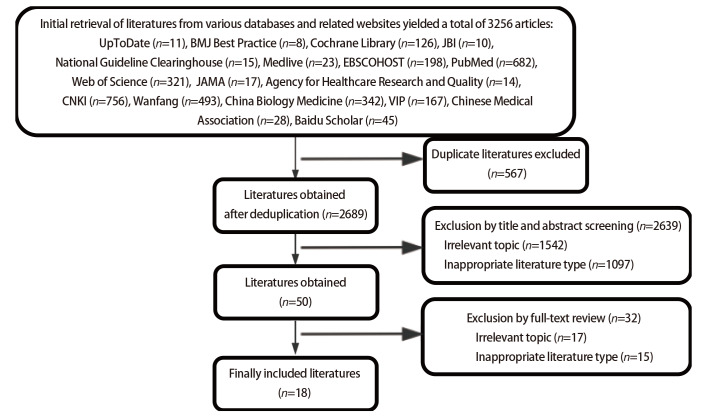
文献筛选流程

**表1:** 纳入文献的基本特征

Author	Literature theme	Literature type
Chambers DC, et al.^[1]^	International organ transplant registry thoracic report	Cohort study
Respiratory Nursing Committee of Zhejiang Nursing Association^[2]^	Expert consensus on postoperative pulmonary rehabilitation nursing for lLTRs	Expert consensus
Gao SH, et al.^[3]^	Observation on the efficacy of pulmonary rehabilitation training in the rehabilitation process of recipients after lung transplantation	Quasi-experimental study
Thoracic Surgery Society of the Shanghai Medical Association, et al.^[4]^	Expert consensus on lung transplantation evaluation for patients with respiratory failure caused by viral lung injury	Expert consensus
Wang XJ, et al.^[5]^	Expert consensus on nursing cooperation in lung transplantation surgery (2022 version)	Expert consensus
Chen RY, et al.^[6]^	Evidence summary of exercise training in LTRs	Evidence summary
Zheng SH, et al.^[7]^	Application of early nutrition and rehabilitation training in LTRs	Quasi-experimental study
Da L, et al.^[8]^	Early pulmonary rehabilitation in ARDS patients	Cohort study
Zhou SF, et al.^[9]^	Application of integrated medical-nursing-rehabilitation management model in pulmonary rehabilitation of LTRs	Quasi-experimental study
Pan YY, et al.^[10]^	Application effect of multifunctional rehabilitation nursing bed in rehabilitation training of LTRs	RCT
Liang JSY, et al.^[11]^	Construction and application of a matching exercise training program for LTRs	Quasi-experimental study
Chen RY, et al.^[12]^	Summary of the best evidence on physical activity in LTRs during hospitalization	Evidence summary
Silva MMCD, et al.^[13]^	Responders COPD patients to two different home-based rehabilitation programs	RCT
Wang YR, et al.^[14]^	Summary of evidence on perioperative nutrition management in LTRs	Evidence summary
Cao XD, et al.^[15]^	Effect of medication self-management program training on medication adherence, pulmonary function, and social function in LTRs	Quasi-experimental study
Luo H, et al.^[16]^	Effect of early quantitative pulmonary rehabilitation on pulmonary function in LTRs	RCT
Machado A, et al.^[17]^	Short-term effects of home-based pulmonary rehabilitation	RCT
Xia QY, et al.^[18]^	Summary of the best evidence on home self-management for LTRs	Evidence summary

LTRs: lung transplant recipients; ARDS: acute respiratory distress syndrome; COPD: chronic obstructive pulmonary disease; RCT: randomized controlled trial.

### 2.2 文献质量评价结果

#### 2.2.1 专家共识

研究共纳入3篇专家共识^[2,4,5]^。经AGREE II评价，所有专家共识在各领域标准化百分比得分中，范围和目的90%以上，参与人员80%以上，严谨性85%以上，清晰性90%以上，应用性80%以上，独立性85%以上。所有专家共识均为A级，予以纳入（表2）。

**表2:** AGREE II评分结果

Author	Standardized percentage by field						Number of fields with ≥30% (*n*)	Number of fields with <30% (*n*)	Overall recommendation grading
	Scope and purpose	Participants	Rigor	Clarity	Applicability	Independence			
Respiratory Nursing Committee of Zhejiang Nursing Association^[2]^	93%	85%	88%	94%	83%	86%	6	0	A
Thoracic Surgery Society of the Shanghai Medical Association, et al.^[4]^	92%	83%	89%	95%	81%	87%	6	0	A
Wang XJ, et al.^[5]^	91%	82%	87%	93%	80%	85%	6	0	A

#### 2.2.2 系统评价/证据总结

本研究共纳入4篇系统评价/证据总结^[6,12,14,18]^，均符合10条以上质量评价标准，整体质量较高，予以纳入（表3）。

**表3:** 系统评价/证据总结质量评价

Author	Clear research question	Appropriate inclusion criteria	Comprehensive search strategy	Quality assessment of included studies	Appropriate data extraction method	Appropriate data synthesis method	Publication bias assessment	Declaration of conflicts of interest	Overall quality evaluation
Chen RY, et al.^ [12]^	Yes	Yes	Yes	Yes	Yes	Yes	Yes	Yes	High
Xia QY, et al.^ [18]^	Yes	Yes	Yes	Yes	Yes	Yes	Yes	Yes	High
Chen RY, et al.^[6]^	Yes	Yes	Yes	Yes	Yes	Yes	Yes	Yes	High
Wang YR, et al.^[14]^	Yes	Yes	Yes	Yes	Yes	Yes	No	Yes	High

The quality assessment was conducted using the evaluation criteria from the Joanna Briggs Institute (JBI) in Australia.

#### 2.2.3 随机对照试验与类实验研究

本研究共纳入4篇随机对照试验^[10,13,16,17]^和5篇类实验研究^[3,7,9,11,15]^，采用Cochrane偏倚风险评估工具评价，所有研究偏倚风险较低或中等，质量满足要求，予以纳入。

**表4:** 队列研究质量评价

Literature authors	Selection (0-4 points)	Comparability (0-2 points)	Outcome (0-3 points)	Total score (0-9 points)	Quality evaluation
Chambers DC, et al.^[1]^	4	2	3	9	High-quality
Da L, et al.^[8]^	4	2	2	8	High-quality

Total score of ≥7 indicates high-quality.

#### 2.2.4 队列研究

本研究共纳入2篇队列研究^[1,8]^，采用Newcastle-Ottawa Scale（NOS）量表评价，所有研究得分均≥7分，质量较高，予以纳入。

### 2.3 证据汇总

通过对LTRs肺功能康复管理的相关文献证据的提取与整合，最终形成LTRs肺功能康复管理的最佳证据共30条，涵盖康复评估、早期干预、运动训练、营养管理、药物管理、呼吸功能训练、心理支持及长期随访8个方面，具体见表5。

**表5:** LTRs肺功能康复管理的最佳证据总结

Theme	Evidence content	Evidence level	Priority
Rehabilitation assessment	1. A comprehensive pulmonary function assessment should be conducted preoperatively, including indicators such as VC, FEV_1_ and TLC.	1	★★★
	2. Preoperative assessment of patients' exercise tolerance is required, and the 6MWT can be used as a core assessment indicator.	1	★★★
	3. Patients' nutritional status should be evaluated preoperatively, including BMI, human serum albumin and total protein.	1	★★★
	4. Postoperative pulmonary function indicators should be monitored regularly, including FEV_1_, FVC and FEV_1_/FVC.	2	★★
	5. Patients' exercise capacity should be assessed regularly, including indicators such as 6MWT, BODE index and modified Barthel index.	2	★★
Early intervention	6. Early rehabilitation intervention should be initiated within 24 h after surgery, including position management and passive activities.	1	★★★
	7. Oxygen therapy management should be implemented in the early postoperative period, and oxygen flow should be adjusted according to blood oxygen saturation.	1	★★★
	8. During the ICU stay, daily rehabilitation assessments should be performed to adjust the rehabilitation plan in a timely manner.	2	★★
	9. Postoperatively, endotracheal intubation should be removed as early as possible to reduce the duration of mechanical ventilation.	2	★★
Exercise training	10. Exercise training should follow the principles of individualization and phased progression, with exercise intensity gradually increased according to the patient’s tolerance.	2	★★
	11. Exercise training should include aerobic exercise and resistance training, conducted at least 3-5 times per week.	1	★★★
	12. Aerobic exercise can be carried out in forms such as walking and cycling, lasting 20-30 min each time.	1	★★★
	13. Exercise intensity should be controlled at 60%-80% of the maximum heart rate to avoid excessive fatigue.	2	★★
	14. A matched exercise plan should be developed based on the patient’s BMI, airway resistance, and degree of dyspnea.	2	★★
Nutrition management	15. Enteral nutrition should be initiated as early as possible after surgery, preferably within 24-48 h.	1	★★★
	16. Sufficient energy intake should be ensured, at a rate of 25-30 kcal/kg•d.	2	★★
	17. Sufficient protein intake should be ensured, at a rate of 1.2-1.5 g/kg•d.	2	★★
	18. Nutritional indicators such as serum albumin and total protein should be monitored regularly.	2	★★
Medication management	19. Patient education on immunosuppressant use should be strengthened to improve medication adherence.	2	★★
	20. The blood concentration of immunosuppressants should be monitored regularly, with timely dosage adjustments.	1	★★
	21. The use of an electronic recording system is recommended to improve patients’ long-term medication adherence.	3	★
Respiratory function training	22. Respiratory muscle training can increase the MIP and respiratory muscle strength in LTRs.	1	★★★
	23. Airway clearance techniques (e.g., effective coughing, back tapping for sputum excretion) are key measures to prevent postoperative pulmonary infections.	1	★★★
	24. Respiratory training should include deep breathing exercises, pursed-lip breathing, and diaphragmatic breathing.	2	★★
	25. Quantitative pulmonary rehabilitation is superior to conventional pulmonary rehabilitation, as it more effectively improves pulmonary function and inflammatory indicators.	2	★★
Psychological support	26. Psychological intervention should be integrated throughout the entire rehabilitation process to alleviate patients’ anxiety and depression.	3	★
	27. Group support and peer education help improve patients’ self-efficacy and rehabilitation adherence.	3	★
Long-term follow-up	28. Home-based rehabilitation training should be continued after discharge, with seamless connection of the hospital-community-family three-level rehabilitation system.	2	★★
	29. Tele-rehabilitation monitoring and guidance can improve patients’ adherence and rehabilitation outcomes.	2	★★
	30. Regular follow-up assessments every 6 months enable timely identification and management of issues during rehabilitation.	2	★★

Explanation of evidence levels: Level 1: Level A evidence (strong recommendation); Level 2: Level B evidence (weak recommendation); Level 3: Level C evidence (conditional recommendation). Priority: ★★★: Core evidence; ★★: Important evidence; ★: Supplementary evidence. Core evidence (★★★) includes 10 items: Evidence 1, 2, 3, 6, 7, 11, 12, 15, 22, 23; Important evidence (★★) includes 16 items: Evidence 4, 5, 8, 9, 10, 13, 14, 16, 17, 18, 19, 20, 24, 25, 28, 29 and 30; Supplementary evidence (★) includes 3 items: Evidence 21, 26 and 27. VC: vital capacity; FEV_1_: forced expiratory volume in one second; TLC: total lung capacity; 6MWT: 6-minute walk test; BMI: body mass index; FVC: forced vital capacity; ICU: intensive care unit; MIP: maximum inspiratory pressure.

## 3 讨论

### 3.1 早期评估与干预是LTRs康复的基础

肺移植术后早期评估与干预是康复成功的关键基础。6分钟步行试验（6-minute walk test, 6MWT）可作为评估LTRs术后运动耐力的核心指标^[6]^。6MWT是评估心肺功能和运动耐力的简便、安全的测试方法，正常成人6MWT为400-700 m。一项实验研究^[7]^表明，早期康复干预可显著缩短机械通气时间、重症监护室（intensive care unit, ICU）停留时间，降低谵妄发生率（*P*<0.05）。Da等^[8]^在急性呼吸窘迫综合征（acute respiratory distress syndrome, ARDS）患者的研究中也证实，早期肺康复干预能够显著改善患者的呼吸功能并降低并发症发生率，这一发现对LTRs同样具有重要指导意义。有研究^[9]^表明通过肺康复早期直接为患者处方氧疗，根据肺功能调整氧疗方式，联合多学科合作实现医护康一体化管理，可显著提升患者术后肺功能指标和日常生活活动能力。同时辅以多功能康复护理床等专业康复设备，在促进LTRs康复和提高护理质量方面具有重要价值^[10]^。

### 3.2 个体化、阶段性的运动训练是康复核心组成部分

运动训练是LTRs肺功能康复的核心内容。一项类实验研究^[11]^表明通过构建匹配式运动训练方案，基于患者体重指数、气道阻力、呼吸困难程度以及运动能力指数分阶段进行运动康复训练，对患者术后早期康复有明显疗效（*P*<0.05），突显了个体化、阶段性训练方案的价值。另外一项针对LTRs住院期间体力活动训练的证据总结研究^[12]^表明LTRs住院期间体力活动对患者术后康复具有重要意义，该研究共汇总形成26条最佳证据，提示临床医护人员应重视LTRs住院期间的体力活动训练，指导临床构建最佳运动方案。Da Silva等^[13]^对慢性阻塞性肺疾病（chronic obstructive pulmonary disease, COPD）患者居家康复训练的随机对照试验比较两种不同居家康复方案的效果，支持定期监督指导患者进行居家康复运动对肺功能康复具有重要意义，该项研究虽然对象为COPD患者，但对肺移植术后患者居家康复训练也具有良好的指导意义。

### 3.3 营养管理与运动训练协同促进肺功能恢复

肺功能的恢复与营养状况及运动能力密切相关，其中营养管理是LTRs康复的重要组成部分。王意茹等^[14]^的证据总结强调，肺移植术后应进行早期肠内营养，保障足够的蛋白质和能量摄入，同时，术后监测人血清白蛋白和总蛋白水平，保证足够的蛋白质摄入，对支持机体恢复和免疫功能加强具有重要意义。术后营养干预与运动训练联合实施可显著提高肺功能恢复和日常活动能力，表明早期营养干预与康复训练相结合，对促进LTRs术后康复进程有明显效果。因此LTRs术后应重视营养管理和运动康复的协同作用，尽早促进肺功能康复。

### 3.4 药物管理与呼吸功能训练对长期预后影响重大

药物管理是LTRs长期预后的关键因素。曹晓东等^[15]^对LTRs服药依从性影响的研究表明，加强患者术后长期用药的管理和健康教育可有效提高患者术后长期用药依从性，对于术后并发症护理、维护移植肺功能具有重要意义，同时还能促进患者更早回归社会。罗红等^[16]^对LTRs术后并发症的随机对照试验证实，加强呼吸功能训练，量化肺康复治疗，可更有效改善肺功能和炎症指标，同时住院时间也明显缩短（*P*<0.05），与常规肺康复治疗相比具有明显优势。Machado等^[17]^进行的一项随机对照试验评估了家庭肺康复训练对COPD急性加重期患者的短期效果。在常规治疗基础上增加3周的家庭肺康复训练，能改善患者的症状和肺功能，对LTRs术后居家康复阶段的治疗方案设计具有重要参考价值，研究采用混合研究方法，综合定量评估和患者自我报告的影响，提供了更全面的证据支持。呼吸肌训练可提高LTRs的最大吸气压（maximum inspiratory pressure, MIP）和呼吸肌力量，气道廓清技术如有效咳嗽、拍背排痰是预防术后肺部感染的关键措施。

### 3.5 长期随访与居家康复是延续照护的重点

夏秋月等^[18]^在一项关于LTRs居家自我管理的研究中指出，出院后居家康复训练应持续进行，医院-社区-家庭三级康复体系无缝衔接至关重要。远程康复监测和指导可提高患者依从性和康复效果，移植后6、12和24个月是评估肺功能恢复和生活质量的关键时间点。这些证据为建立LTRs长期随访体系提供重要依据，有助于确保康复效果的持续性和稳定性。

### 3.6 证据的临床转化策略与实施建议

本研究形成的30条证据为LTRs康复管理提供系统性的循证依据，在临床转化过程中需要考虑以下实施策略：

证据的实施优先级应基于患者安全和康复效果，核心证据如早期康复评估（证据1-3）和早期干预（证据6-7）作为临床实践的基础标准，所有医疗机构均应优先实施，重要证据个体化运动训练方案（证据10-14）需要根据机构资源条件逐步实施，补充证据如电子记录系统（证据21）可根据信息化水平选择性实施。不同医疗机构的资源条件差异显著影响证据的适用性，三级医院可全面实施所有推荐证据，二级医院应重点关注核心证据和部分重要证据，基层医疗机构可重点实施居家康复相关证据（证据28-30）。在实际应用中，医疗机构需要根据自身的人员配置、设备条件和技术水平，制定分阶段、分层次的实施计划，确保证据转化的可行性和有效性。

### 3.7 与国际权威指南的对比分析

本研究结果与美国胸科学会（American Thoracic Society, ATS）肺康复指南和欧洲呼吸学会（European Respiratory Society, ERS）肺移植管理共识具有良好的一致性，在运动训练方面，推荐的有氧训练与抗阻训练相结合的方案（证据11）与ATS指南推荐一致。在营养管理强调的早期肠内营养（证据15）与ERS共识的推荐相符，《中国肺动脉高压诊断与治疗指南（2021版）》^[19]^的相关解读也为LTRs的康复管理提供了重要参考。本研究在某些方面提供更为具体的实施细节，明确运动强度为最大心率的60%-80%（证据13），国际指南多为原则性描述。本研究特别强调匹配式运动训练方案（证据14），在国际指南中较少涉及，体现本研究在个体化康复方案方面的创新性贡献。长期随访方面，本研究提出的医院-社区-家庭三级康复体系（证据28）比国际指南的建议更加系统和具体。

### 3.8 研究局限性与未来研究方向

本研究存在以下局限性：（1）纳入文献数量相对较少（18篇），可能影响证据的代表性和推广性；（2）缺乏对不同移植类型（单肺 *vs* 双肺）、术后不同时间阶段、不同原发疾病类型的亚组分析，限制证据的精准化应用；（3）证据整合过程中对文化差异和医疗体系差异的考虑不足，可能影响证据在不同地区和医疗环境中的适用性。

未来研究应重点关注方向：（1）开展多中心、大样本的随机对照试验，特别是针对不同移植类型和术后时间阶段的比较研究，提供更高质量的循证证据；（2）发展数字化、智能化的康复监测技术，如可穿戴设备监测、人工智能辅助评估等，提高远程康复的精准性和效果；（3）建立个体化康复方案的预测模型，基于患者的基线特征、移植类型、合并症等因素制定精准康复策略；（4）开展卫生经济学评价，为证据的推广应用提供成本效益分析，促进循证实践的可持续发展；（5）加强国际合作，开展跨文化、跨医疗体系的证据验证研究，提高证据的普适性。

总之，研究基于循证医学的原则，系统整合LTRs肺功能康复管理的最佳证据，提出了30条临床应用建议，建议涵盖康复评估、早期干预、运动训练、营养管理、药物管理、呼吸功能训练、心理支持及长期随访8个关键方面。早期评估与干预、个体化阶段性运动训练、营养支持、药物管理、呼吸功能训练和长期随访是LTRs肺功能康复管理的核心要素。多学科协作的医护康一体化模式是LTRs康复管理的有效方式，匹配式运动训练方案和量化肺康复治疗能有效改善患者肺功能和生活质量。术后早期营养干预与运动训练相结合，可促进患者术后快速康复。药物自我处置程式训练对提高患者长期用药依从性和维护移植肺功能具有重要价值。
